# Supplementary material for: Validating the Nepalese Short Attitudes Toward Mental Health Problems Scale (N-SATMHPS): A Culturally Sensitive Tool for Assessing Mental Health Stigma
Source: Int J Environ Res Public Health. 2025 Dec 18;22(12):1884. doi: 10.3390/ijerph22121884 (PMC12732979; doi:10.3390/ijerph22121884)
Supplement: Supplementary file 1 [file ijerph-22-01884-s001.zip › ijerph-3927223-supplementary.pdf]

Nepalese Shorter Version of Attitudes Towards Mental Health Problems Scale (N-SATMHPS)

Dev Bandhu Poudel*, Takashi Yoshioka, Rory Colman, Yasuhiro Kotera

* Correspondence: dvbpoudel@gmail.com

नेपाली छोटो मानसिक स्वास्थ्य समस्याप्रतिको मनोवृत्ति प्रश्नावली

हामी मानसिक स्वास्थ्य समस्याका बारेमा मानिसहरूले के सोच्छन र कसरी लिन्छन, मानसिक स्वास्थ्य समस्यालाई कसरी हेर्छन भन्ने कुरामा जान्न चाहन्छौं । तपाईंलाई थाहै होला, केही मानिसहरू डिप्रेसन (निराशा) र एन्जाइटी (चिन्ता) जस्ता मानसिक स्वास्थ्य समस्याबाट पीडित रहेका हुन्छन । यी समस्याहरूले उनीहरूको लागि दैनिक जीवनमा सहजरुपमा अघि बढ्न वा दैनिक जीवनको सामना गर्न कठिनाइ उत्पन्न गराउन सक्छन । डिप्रेसन भएका मानिसहरू थकित महसुस गर्न सक्छन, जीवनमा रमाउन सक्दैनन, आफूलाई एकान्तमा राख्न चाहन्छन र पारिवारिक जीवनदेखि पर बस्ने हुन सक्छन । तल केही भनाइरकथनहरू छन जुन तपाईंले, तपाईंको समुदायले वा तपाईंको परिवारले मानसिक स्वास्थ्यसँग सम्बन्धित समस्यालाई कसरी हेर्ने गरेको छ भन्ने देखाउँछ । हरेक कथन ध्यानपूर्वक पढी ती कथनहरूसँग तपाईं कत्तिको समहत हुनुहुन्छ भन्ने देखाउन उपयुक्त कुनै एउटा अंकमा गोलो घेरा लगाउनुहोस।

कृपया तलको मापनस्तर प्रयोग गर्नुहोस

० . पटक्कै सहमत छैन १ . अलिअलि सहमत २ . धैरै हदसम्म सहमत ३ . पूर्ण सहमत

यहाँ कोष्ठमा भएको अंकले मूल प्रश्नावलीको प्रश्न नम्बरलाई जनाउँछ।

**मानसिक स्वास्थ्यप्रतिको मनोभाव**					
प्रश्नहरूको यस पहिलो समूहको लागि, दैनिक जीवनको सामना गर्न कठिन बनाउने डिप्रेसन र एन्जाइटी जस्ता मानसिक स्वास्थ्य समस्यालाई तपाईंको समुदाय र परिवारले कसरी हेर्छन भन्नेबारे केहीबेर सोचविचार गरी तलको कुनै उपयुक्त अंकमा गोलो घेरा लगाउनुहोस ।					
**समुदायको मनोवृत्ति**					
१. (२)	मेरो समुदायले मानसिक स्वास्थ्य समस्यालाई व्यक्तिगत कमजोरीको रुपमा हेर्छ ।	०	१	२	३
२. (३)	मेरो समुदायमा मानसिक स्वास्थ्य समस्या भएको मानिसलाई तिरस्कारको नजरले हेरिन्छ ।	०	१	२	३
**पारिवारिक मनोवृत्ति**					
३. (७)	मेरो परिवारमा मानसिक स्वास्थ्य समस्या भएको मानिसलाई तिरस्कारको नजरले हेरिन्छ ।	०	१	२	३
४. (८)	मेरो परिवार मानसिक स्वास्थ्य समस्या भएका मानिसहरूबाट पर बस्न चाहन्छ ।	०	१	२	३
**बाहिरी लाज**					
अब अर्को प्रश्नहरूको समूहको लागि, दैनिक जीवनको सामना गर्न कठिन बनाउने डिप्रेसन र एन्जाइटी जस्ता मानसिक स्वास्थ्य समस्याबाट यदि तपाईं पिडित हुनुभएमा तपाईंलाई तपाईंको समुदाय वा परिवारले कसरी हेर्छ होला, केहीबेर सोचविचार गरी तलका कुनै उपयुक्त अंकमा गोलो घेरा लगाउनुहोस ।					

**सामुदायिक बाहिरी लाज**					
५. (११)	मलाई लाग्छ मेरो समुदायले मलाई काम नलाग्ने मानिसको रुपमा हेर्नेछ ।	०	१	२	३
६. (१२)	मलाई लाग्छ मेरो समुदायले मलाई कमजोर मानिसको रुपमा हेर्नेछ ।	०	१	२	३
**पारिवारिक बाहिरी लाज**					
७. (१५)	मलाई लाग्छ मेरो परिवारले मलाई निकृष्ट (तल्लो) व्यक्तिको रुपमा हेर्नेछ ।	०	१	२	३
८. (१६)	मलाई लाग्छ मेरो परिवारले मलाई काम नलाग्ने मानिसको रुपमा हेर्नेछ ।	०	१	२	३
**आन्तरिक लाज**					
अब अर्को प्रश्नहरूको समूहको लागि, दैनिक जीवनको सामना गर्न कठिन बनाउने डिप्रेसन र एन्जाइटी जस्ता मानसिक स्वास्थ्य समस्याबाट तपाईं पिडित हुनुभएको अवस्थामा तपाईं आफूलाई कस्तो महसुस हुन्छ होला, केहीबेर सोचविचार गरी तलका कुनै उपयुक्त अंकमा गोलो घेरा लगाउनुहोस ।					
९. (१९)	म आफूलाई तल्लो व्यक्तिको रुपमा हेर्नेछु ।	०	१	२	३
१०. (२०)	म आफूलाई काम नलाग्ने व्यक्तिको रुपमा हेर्नेछु ।	०	१	२	३
**परिवार प्रतिबिम्बित लाज**					
अब अर्को प्रश्नहरूको समूहको लागि, दैनिक जीवनको सामना गर्न कठिन बनाउने डिप्रेसन र एन्जाइटी जस्ता मानसिक स्वास्थ्य समस्याबाट तपाईं पिडित हुनुभएको अवस्थामा तपाईं कस्तो महसुस हुन्छ होला । यसपटक ती समस्याका कारण तपाईंको परिवारमा पर्ने प्रभावबारे तपाईं कत्तिको चिन्तित हुनुहुनेछ, केहीबेर सोचविचार गरी तलका कुनै उपयुक्त अंकमा गोलो घेरा लगाउनुहोस ।					
११. (२४)	मेरो परिवारलाई तल्लो रुपमा हेरिनेछ ।	०	१	२	३
१२. (२५)	मेरो परिवारलाई काम नलाग्ने रुपमा हेरिनेछ ।	०	१	२	३
**आत्म प्रतिबिम्बित लाज**					
अब अर्को प्रश्नहरूको समूहको लागि, दैनिक जीवनको सामना गर्न कठिन बनाउने डिप्रेसन र एन्जाइटी जस्ता मानसिक स्वास्थ्य समस्याबाट तपाईंको कुनै नजिकको नातेदार पिडित हुनुभएको अवस्थामा तपाईं कस्तो महसुस हुन्छ होला । यसपटक ती समस्याका कारण तपाईंमा पर्ने प्रभावबारे तपाईं कत्तिको चिन्तित हुनुहुनेछ, केहीबेर सोचविचार गरी तलका कुनै उपयुक्त अंकमा गोलो घेरा लगाउनुहोस ।					
१३. (३३)	मेरो आफ्नै मानसम्मानमा हानि पुग्ला कि भनेर म चिन्तित हुनेछु ।	०	१	२	३
१४. (३४)	यदि यो कुरा थाहा भयो भने मेरो समुदायमा मेरो प्रतिष्ठा घट्नेछ भनेर मलाई चिन्ता लाग्नेछ ।	०	१	२	३

नेपाली छोटो मानसिक स्वास्थ्य समस्याप्रतिको मनोवृत्ति प्रश्नावली (N-SATMHPS) मूल्याङ्कन म्यानुअल (नेपाली संस्करण)

मूल्याङ्कन (Scoring)

हरेक उप-आयामका कथनहरूको स्कोरहरूलाई जोडेर पूर्ण-स्कोर निकालिन्छ।

विवरण

अघिल्लो अनुसन्धानले Gilbert et al. (2004) द्वारा एसियाली महिलाहरूमा मानसिक स्वास्थ्य समस्यासँग सम्बन्धित लाजका विभिन्न सांस्कृतिक मनोवृत्तिहरू उजागर गरेको थियो, जुन समुदायका मनोवृत्ति, परिवारका मनोवृत्ति, र आत्म-मनोवृत्तिसँग सम्बन्धित छन्। यी मनोवृत्तिहरूले विभिन्न प्रकारको बाह्य लाज (external shame)—व्यक्तिहरूले अरूले आफूलाई कसरी मूल्याङ्कन गर्ने भन्ने कुरामा केन्द्रित हुने—साथै मानसिक स्वास्थ्य समस्यासँग सम्बन्धित व्यक्तिगत (आन्तरिक) लाज-केन्द्रित मनोवृत्तिहरूलाई पनि दर्शाउँछन्। यसबाहेक, यसले प्रतिबिम्बित लाज (reflected shame) समावेश गर्दछ, जसमा आफ्नो कारणले परिवारका सदस्यमा हुने लाज (परिवार-प्रतिबिम्बित लाज) र परिवारको सदस्यको कारणले आफूलाई लाग्ने लाज (आत्म-प्रतिबिम्बित लाज) दुबै समावेश छन्, साथै सांस्कृतिक अवधारणा इज्जत (Izzat) पनि समेटिएको छ। यी प्रक्रियाहरू नेपाल संदर्भमा अत्यन्त सान्दर्भिक छन् (Gilbert et al., 2004; Gilbert & Andrews, 1998; Gilbert & Miles, 2002).

यसलाई सम्बोधन गर्न, हामीले मूल ३५-आइटमको 'नेपाली छोटो मानसिक स्वास्थ्य समस्याप्रतिको मनोवृत्ति प्रश्नावली' (ATMHP; Gilbert et al., 2007) को छोटो नेपाली संस्करण विकास गर्यौं। नेपाली छोटो मानसिक स्वास्थ्य समस्याप्रतिको मनोवृत्ति प्रश्नावली (N-SATMHPS) मा १४ विशेष रूपमा चयन गरिएका आइटमहरू छन्, जसले पाँच खण्डमा सात आयाम समेट्छन् र मूल स्केलको संरचना र उद्देश्यलाई पूर्ण रूपमा कायम राख्छन्।

खण्ड १: मनोवृत्ति (Attitudes)

१. सामुदायिक मनोवृत्ति (Community Attitudes)

– मानसिक स्वास्थ्य समस्यालाई समुदायले कसरी हेर्छ भन्ने व्यक्तिको बुझाइ (प्रश्न १–२; α = .७४; r = .९४)

२. पारिवारिक मनोवृत्ति (Family Attitudes)

– मानसिक स्वास्थ्य समस्याबारे परिवारको धारणा के हो भन्ने व्यक्तिको बुझाइ (प्रश्न ३–४; α = .८४; r = .९१)

खण्ड २: बाहिरी लाज (External Shame)

३. सामुदायिक बाहिरी लाज (Community External Shame)

– मानसिक स्वास्थ्य समस्या भएमा समुदायले आफूलाई कसरी हेर्नेछ भन्ने विश्वास (प्रश्न ५–६; α = .९१; r = .९६)

४. पारिवारिक बाहिरी लाज (Family External Shame)

– मानसिक स्वास्थ्य समस्या भएमा परिवारले आफूलाई कसरी हेर्नेछ भन्ने विश्वास (प्रश्न ७–८; α = .९२; r = .९६)

खण्ड ३: आन्तरिक लाज (Internal Shame)

५. आन्तरिक लाज (Internal Shame)

– मानसिक स्वास्थ्य समस्या हुनुका कारणले उत्पन्न आत्म-आलोचनात्मक वा नकारात्मक आत्म-मूल्यांकन (प्रश्न ९–१०; α = .८९; r = .९०)

खण्ड ४: परिवार प्रतिबिम्बित लाज (Reflected Shame – Family)

६. परिवार प्रतिबिम्बित लाज (Family Reflected Shame)

– आफूलाई मानसिक स्वास्थ्य समस्या परेमा आफ्नो परिवारलाई समाजले कसरी हेर्नेछ भन्ने विश्वास (प्रश्न ११–१२; α = .९१; r = .८५)

खण्ड ५: आत्म प्रतिबिम्बित लाज (Reflected Shame – Self)

७. आत्म प्रतिबिम्बित लाज (Self-Reflected Shame)

– नजिकका पारिवारिक सदस्यलाई मानसिक स्वास्थ्य समस्या भएमा आफ्नो प्रतिष्ठा वा सामाजिक मान-सम्बानमा नकरात्मक प्रभाव पर्न सक्ने चिन्ता (प्रश्न १३–१४; α = .८९; r = .९५)

उत्तर दिने तरिका (Response pattern)

सबै १४ प्रश्नहरू ४-बिन्दु लिकर्ट स्केलमा आधारित छन्: “०” = “पटक्कै सहमत छैन” देखि “३” = “पूर्ण सहमत”

कुल स्कोर ० देखि ४२ सम्म हुन सक्छ, र उच्च स्कोरले मानसिक स्वास्थ्य समस्याप्रतिको बढी नकारात्मक मनोवृत्ति वा बढी लाज/कलंक सम्बन्धी चिन्ता जनाउँछ।
